# A Comparative Metabolomic Evaluation of Behcet’s Disease with Arthritis and Seronegative Arthritis Using Synovial Fluid

**DOI:** 10.1371/journal.pone.0135856

**Published:** 2015-08-13

**Authors:** Joong Kyong Ahn, Sooah Kim, Jungyeon Kim, Jiwon Hwang, Kyoung Heon Kim, Hoon-Suk Cha

**Affiliations:** 1 Department of Internal Medicine, Division of Rheumatology, Kangbuk Samsung Hospital, Sungkyunkwan University School of Medicine, Seoul, Republic of Korea; 2 Department of Biotechnology, Graduate School, Korea University, Seoul, Republic of Korea; 3 Department of Medicine, Division of Rheumatology, Samsung Medical Center, Sungkyunkwan University School of Medicine, Seoul, Republic of Korea; The Norwegian University of Science and Technology (NTNU), NORWAY

## Abstract

Behcet’s disease (BD) with arthritis is often confused with seronegative arthritis (SNA) because of shared clinical symptoms and the lack of definitive biomarkers for BD. To investigate possible metabolic patterns and potential biomarkers of BD with arthritis, metabolomic profiling of synovial fluid (SF) from 6 patients with BD with arthritis and 18 patients with SNA was performed using gas chromatography/time-of-flight mass spectrometry in conjunction with univariate and multivariate statistical analyses. A total of 123 metabolites were identified from samples. Orthogonal partial least square-discriminant analysis showed clear discrimination between BD with arthritis and SNA. A set of 11 metabolites were identified as potential biomarkers for BD using variable importance for projection values and the Wilcoxon-Mann-Whitney test. Compared with SNA, BD with arthritis exhibited relatively high levels of glutamate, valine, citramalate, leucine, methionine sulfoxide, glycerate, phosphate, lysine, isoleucine, urea, and citrulline. There were two markers identified, elevated methionine sulfoxide and citrulline, that were associated with increased oxidative stress, providing a potential link to BD-associated neutrophil hyperactivity. Glutamate, citramalate, and valine were selected and validated as putative biomarkers for BD with arthritis (sensitivity, 100%; specificity, 61.1%). This is the first report to present potential biomarkers from SF for discriminating BD with arthritis from SNA. The metabolomics of SF may be helpful in searching for potential biomarkers and elucidating the clinicopathogenesis of BD with arthritis.

## Introduction

Behcet’s disease (BD) is a chronic, complex systemic vasculitis of unknown etiology characterized by orogenital ulcers, uveitis, and arthritis, which is more prevalent in Korea, China, Japan, and Turkey [1]. Genetic and environmental factors, immunologic abnormalities, and endothelial dysfunction appear to play important roles in the pathogenesis of BD. At present, there are many aspects which are yet to be solved in BD, for example the absence of specific diagnostic tests and unknown etiopathogenesis. As a practical example, it may be impossible to distinguish intestinal BD from Crohn's disease.

Arthritis and arthralgias in BD are known to be the most common rheumatologic findings with a prevalence ranging from 40 to 70% [2]. When joint manifestations antedate other features of BD by months or years, it becomes difficult to differentiate BD with arthritis from other types of inflammatory arthritis. Asymmetric mono-oligoarthritis, erythema nodosum, and uveitis have been observed in both BD with arthritis and seronegative spondyloarthropathy (SNSpA) [2–4]. Additionally, enthesopathy, the typical pathology of spondyloarthropathy, is associated with BD with acne/arthritis [5]. The involvement of wrists and elbows with a sub-acute or chronic course, and even a symmetrical fashion in BD may mimic seronegative rheumatoid arthritis (SNRA) [6]. There are no pathologic findings from synovial tissue or synovial fluid (SF) analysis that allow for discrimination between BD with arthritis and early rheumatoid arthritis (RA) [7,8]. Collectively, it is difficult to clinically distinguish BD with arthritis from seronegative arthritis (SNA), such as SNSpA or SNRA, due to an overlap of clinical symptoms. Therefore, it may possible to improve treatment results and avoid unnecessary therapies through an accurate differential diagnosis between BD with arthritis and SNA. However, there are no definite biomarkers to help narrow down a differential diagnosis or pathogenic mechanism of BD with arthritis.

Metabolomics can provide comprehensive quantitative measurements of endogenous metabolites within biological systems [9]. The global metabolomic profiles found in cells, tissues, or biofluids allow for further understanding of the underlying molecular mechanisms of pathophysiological processes and identification of diagnostic biomarkers for complex diseases [10,11]. Metabolomic investigation has recently been used to analyze metabolites from biological fluids to reflect various rheumatic diseases including RA, osteoarthritis, or systemic lupus erythematosus [10–12]. Synovial fluid is a viscous body fluid found in the cavities of synovial joints, which may reflect pathologic changes such as alterations of the inflammatory cytokine and growth factor environment in the setting of inflammatory arthritis. Until now, no investigations using metabolomics have been attempted to improve the diagnosis of and identify potential biomarkers for BD with arthritis using SF. Therefore, we hypothesized that metabolomic profiling of SF could be used during differential diagnosis and could improve understanding of the pathogenic mechanism of BD with arthritis. The purpose of this study was to evaluate the metabolomic profiling of SF for use in differential diagnosis and the specificity of metabolic changes in patients with BD with arthritis compared to those with SNA using a gas chromatography/time-of-flight mass spectrometry (GC/TOF MS)-based metabolomics platform.

## Materials and methods

### Patient samples

All 24 patient samples were drawn from the rheumatology clinic at the Samsung Medical Center and Kangbuk Samsung Hospital in Seoul, Korea. Of those samples, 6 patients (6 males with a mean age ± standard deviation (SD) of 34.8 ± 16.4 years) had BD with arthritis, 13 patients (8 males and 5 females with a mean age ± SD of 30.9 ± 11.0 years) had SNSpA, and 5 patients (1 male and 4 females with a mean age ± SD of 65.8 ± 10.7 years) had SNRA. They met all recruitment criteria following the criteria of the 1990 International Study Group for BD [13], the Assessment of SpondyloArthritis International Society classification criteria for axial spondyloarthritis [14], and the 1987 American College of Rheumatology classification criteria for RA [15], respectively.

Joint effusions from patients were aspirated as clinically indicated using a standard sterile procedure. Final diagnoses were made by experienced rheumatologists. SF samples were collected and stored at -80°C. The experimental protocols used in this study were approved by the Samsung Medical Center (#2014-01-082) and Kangbuk Samsung Hospital institutional review board (#KBC 14082) and written informed consent was obtained from each patient included in this study. This study was conducted in accordance with the principles expressed in the Helsinki Declaration.

### Metabolite sample preparation from synovial fluid

Metabolite samples were prepared from SF using a previously published method with a slight modification [16]. Briefly, SF samples were centrifuged at 500 × *g* at 4°C for 5 min, and the supernatant was collected. The supernatant was mixed with 80% (v/v) methanol at -20°C, and the mixture was vortexed for 3 min at room temperature. The extract was centrifuged at 16,100 × *g* at 4°C for 5 min, and the supernatant was then concentrated to complete dryness in a vacuum concentrator (Labconco, Kansas City, MO). The dried samples were kept at -80°C until derivatization and GC/TOF MS analysis.

### GC/TOF MS analysis

As previously described [16] for GC/TOF MS analysis, the dried metabolites were derivatized with 5 μl of 40 mg/ml of methoxyamine hydrochloride in pyridine (Pierce, Rockford, IL) at 30°C for 90 min and 45 μl of *N*-methyl-*N*-(trimethylsilyl) trifluoroacetamide (Fluka, Buchs, Switzerland) at 37°C for 30 min. A mixture of fatty acid methyl esters (from C8 to C30) was added to the samples as internal retention index markers. GC/TOF MS analysis was conducted using an Agilent 7890B GC (Agilent Technologies, Wilmington, DE) equipped with a Pegasus HT TOF MS (Leco, St. Joseph, MI). For the separation of compounds in metabolite samples, an RTX-5Sil MS capillary column (30 m × 25 mm, 0.25 μm film thickness; Restek, Bellefonte, PA) with an additional 10-m long integrated guard column was used. Then, 1 μl of sample was injected into the GC in a splitless mode with oven temperature held at 50°C for 1 min, which was increased to 330°C at 20°C/min and held for 5 min. Mass spectra of metabolites were acquired in a mass range of 85 to 500 *m/z* at an acquisition rate of 17 spectra/s. The ionization mode was subjected to electron impact at 70 eV and the temperature for the ion source was set to 250°C. The transfer line was set to 250°C and 280°C, respectively.

### Statistical analyses of metabolomic data

Preprocessed data obtained from the Leco Chroma TOF software (ver. 4.50; Leco) were processed using BinBase, an in-house database [16–18]. The peak intensity of the identified metabolites was normalized by the median of the sum of the peak intensities of all identified metabolites in each sample. STATISTICA (ver. 7.1; StatSoft, Tulsa, OK) was used for univariate analyses such as principal component analysis (PCA), nonparametric Wilcoxon-Mann-Whitney test, and breakdown and the one-way analysis of variance (ANOVA) [18,19]. Orthogonal partial least square-discriminative analysis (OPLS-DA) and seven-fold internal cross validation was performed using SIMCA-P+ (version 12.0; Umetric AB, Umea, Sweden). MedCalc software (Broekstraat, Mariakerke, Belgium) was used to obtain a receiver operating characteristic (ROC) curve for further evaluation of the diagnostic properties of identified metabolites.

## Results

### Synovial fluid metabolite profiles between BD with arthritis and SNA groups

We performed global metabolite profiling of SF samples from 24 patients with BD (*n* = 6), SNSpA (*n* = 13), and SNRA (*n* = 5) by GC/TOF MS. A total of 123 different metabolites from all samples were identified by BinBase. The higher number of identified metabolites in this study reduces the potential for bias in biological interpretation of metabolic results. The detected metabolites were classified into the following chemical classes: amino acids (23% of the identified metabolites), sugars and sugar alcohols (19%), organic acids (19%), fatty acids (19%), amines (9%), phosphates (8%), and miscellaneous (6%) (Table 1). Additionally, these metabolites were major intermediates of cellular metabolic pathways such as the glycolysis, the TCA cycle, biosynthesis of amino acids and fatty acids, and the urea cycle.

**Table 1:** Identification of 123 metabolites from synovial fluid samples of 24 patients with Behcet’s disease with arthritis and seronegative arthritis using BinBase.

Class	Metabolites	
Amino acids	alanine	lysine
	β-alanine	methionine
	asparagine	methionine sulfoxide
	aspartate	*N*-methylalanine
	citrulline	ornithine
	cyano-L-alanine	oxoproline
	cysteine	phenylalanine
	glutamate	proline
	glutamine	saccharopine
	glycine	serine
	histidine	threonine
	homoserine	tryptophan
	isoleucine	tyrosine
	leucine	valine
Sugars and sugar alcohols	3,6-anhydro-D-galactose	mannose
	arabitol	myo-inositol
	cellobiose	palatinitol
	fructose	raffinose
	galactinol	ribose
	galactose	sucrose
	glucose	tagatose
	glycerol	threitol
	lactulose	threose
	levoglucosan	trehalose
	lyxose	xylose
	mannitol	
Organic acids	adipate	2-hydroxyvalerate
	aminomalonate	2-ketoadipate
	5-aminovalerate	lactate
	benzoate	malate
	citramalate	oxalate
	citrate	phenylacetate
	fumarate	3-phenyllactate
	galactonate	pyruvate
	glycerate	succinate
	glycolic acid	terephthalate
	β-hydroxybutyrate	urate
	3-hydroxypropionate	
Fatty acids	arachidic acid	1-monopalmitin
	arachidonic acid	1-monostearin
	behenic acid	myristic acid
	capric acid	octadecanol
	heptadecanoic acid	oleic acid
	lanosterol	palmitic acid
	lauric acid	palmitoleic acid
	lignoceric acid	pelargonic acid
	linoleic acid	pentadecanoic acid
	linolenic acid	stearic acid
Amines	adenosine	spermidine
	ethanolamine	thymine
	guanine	uracil
	inosine	uridine
	5-deoxy-5-methylthioadenosine	xanthine
	putrescine	
Phosphates	adenosine-5-monophosphate	phosphate
	cytidine-5-monophosphate	phosphogluconate
	glucose-6-phosphate	ribose-5-phosphate
	glycerol-1-phosphate	ribulose-5-phosphate
	inosine-5-monophosphate	trehalose-6-phosphate
Miscellaneous	carnitine	sulfuric acid
	cholic acid	taurine
	salicylaldehyde	α-tocopherol
	squalene	urea

**Fig 1:**
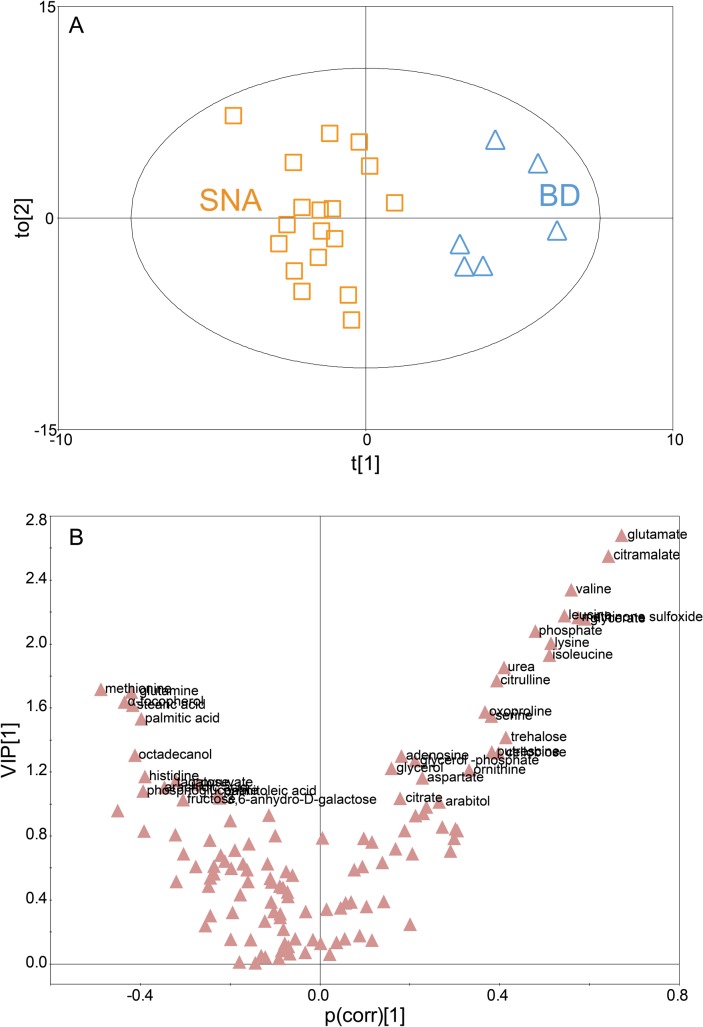
The orthogonal least square-discriminative analysis (OPLS-DA) of metabolomic profiles of synovial fluids of Behcet’s disease (BD) with arthritis and seronegative arthritis (SNA). (A) The score plot of the OPLS-DA model for the BD with arthritis and SNA groups (t[1], score of the non-orthogonal component; to[2], score of the orthogonal component). The generated explained variation values, 0.45 of *R* ^*2*^ *X* and 0.91 of *R* ^*2*^ *Y*, and the predictive capability, 0.64 of *Q* ^*2*^ indicated the excellence in modeling and prediction of the OPLS-DA model, respectively, with clear discrimination between BD with arthritis and SNA groups. (B) V-plot with p(corr) and VIP values of 123 different metabolites in OPLS-DA. The metabolites with p(corr) < 0 were those decreased in the BD with arthritis group while the metabolites with p(corr) > 0 were those increased in the BD with arthritis group. The metabolites with VIP > 1 were represented in Fig 1B.

To determine whether it was possible to distinguish between metabolic profiles of BD with arthritis and SNA using processed data from the GC/TOF MS, PCA and OPLS-DA were carried out in this study. Initially, the PCA was used to investigate general interrelations including grouping, clustering, and outliers among the observations. The score plot of PCA with 0.40 of *R* ^*2*^ *X* and 0.41 of *Q* ^*2*^ indicated that the metabolite profiles of BD and SNA were not significantly discriminated (S1 Fig). Alternatively, OPLS-DA was employed to successfully minimize the possible contribution of intergroup variability and to further increase the difference of metabolic profiles between the two groups (i.e., BD and SNA) comparing with the results from PCA. The metabolite profiles of BD with arthritis and SNA were distinctly separated on the score plot of OPLS-DA (Fig 1A). An OPLS-DA model (one predictive component and two orthogonal components) with 0.45 of *R* ^*2*^ *X*, 0.91 of *R* ^*2*^ *Y*, and 0.64 of *Q* ^2^ was achieved, which indicates superior function of the OPLS-DA model of BD with arthritis and SNA.

### Identification of biomarkers for Behcet’s disease with arthritis

In an effort to identify the putative metabolites that can account for the differentiation of BD with arthritis and SNA groups, multivariate statistical analyses were performed. First, variable importance in projection (VIP) values were obtained from the OPLS-DA model. In VIP analysis, VIP values above 1 are considered important [20]. We selected 37 metabolites with a VIP value greater than 1. Of these 37 metabolites, the levels of 23 metabolites were higher in the BD group while the levels of 14 metabolites were higher in the SNA groups (Fig 1B and S1 Table). Next, the nonparametric Wilcoxon-Mann-Whitney test and breakdown and the ANOVA with post hoc Tukey's honestly significant difference test were performed using STATISTICA for further evaluation of the selected metabolites with high VIP values as candidate biomarkers for BD with arthritis. Using the nonparametric Wilcoxon-Mann-Whitney test, 26 metabolites were eliminated from the 37 candidate biomarkers because their levels were not significantly different between the two groups at a 95% confidence interval. As a result, 11 selected metabolites, which met the criteria of VIP, *P*-value, and area under the ROC curve (AUC) are listed in Table 2. The 11 metabolites showing significantly different abundances between BD with arthritis and SNA included glutamate, citramalate, valine, leucine, methionine sulfoxide, glycerate, phosphate, lysine, isoleuicine, urea, and citrulline.

**Table 2:** VIP and AUC values of synovial fluid metabolites to be used as potential biomarkers for discriminating Behçet's disease with arthritis from seronegative arthritis.

Metabolite	BD vs. SNA			BD vs. SNRA			BD vs. SNSpA		
	VIP	AUC	*P*-value	VIP (rank)	AUC	*P*-value	VIP (rank)	AUC	*P*-value
Glutamate	2.68	0.870	0.001	2.01 (4)	1.000	0.001	2.38 (1)	0.821	0.010
Citramalate	2.55	0.861	0.002	1.93 (5)	1.000	0.002	2.24 (2)	0.808	0.017
Valine	2.34	0.833	0.006	1.79 (9)	1.000	0.006	2.02 (3)	0.769	0.034
Leucine	2.18	0.815	0.012				1.90 (5)	0.769	0.048
Methionine sulfoxide	2.17	0.815	0.012	2.24 (1)	1.000	< 0.001			
Glycerate	2.16	0.796	0.013	1.80 (8)	0.967	0.006			
Phosphate	2.08	0.815	0.017						
Lysine	2.01	0.806	0.022				1.94 (4)	0.782	0.043
Isoleucine	1.93	0.787	0.028						
Urea	1.85	0.852	0.036						
Citrulline	1.77	0.787	0.046						
Glutamine				2.19 (2)	1.000	< 0.001			
Oxoproline				2.11 (3)	1.000	< 0.001			
Threonine				1.88 (6)	1.000	0.003			
Methionine				1.84 (7)	0.900	0.004			
3,6-anhdyro-D-galatose				1.79 (10)	0.967	0.006			
Capric acid				1.75 (11)	0.967	0.008			

AUC, Area under the receiver operator characteristics curve; BD, Behcet’s disease with arthritis; SNA, Seronegative arthritis; SNRA, Seronegative rheumatoid arthritis; SNSpA, seronegative spondyloarthropathy; VIP, variable importance in projection. *P*-values were determined using the Wilcoxon-Mann-Whitney test.

To further select putative biomarkers for BD with arthritis against SNSpA and SNRA, which were each in the SNA group, one-way ANOVA was performed. We found 5 metabolites to be potential biomarkers that exhibited different levels between BD with arthritis and SNSpA: glutamate, citramalate, valine, lysine, and leucine (Table 2). Also, 11 metabolites were identified as potential biomarkers with different levels between BD with arthritis and SNRA: methionine sulfoxide, glutamine, oxoproline, glutamate, citramalate, threonine, methionine, glycerate, valine, 3,6-anhydro-D-galactose, and capric acid (Table 2). Of the metabolites selected as putative biomarkers for differentiating between BD with arthritis and SNSpA or SNRA, there are three common metabolites with high VIP. In order of VIP values from greatest to least, they are glutamate, citramalate, and valine. The fold increases of these metabolites in the BD group ranged from 2.1 to 6.2 (S2 Fig). Finally, we selected three metabolites as putative biomarkers for BD with arthritis against SNSpA or SNRA.

### Model validation

**Fig 2:**
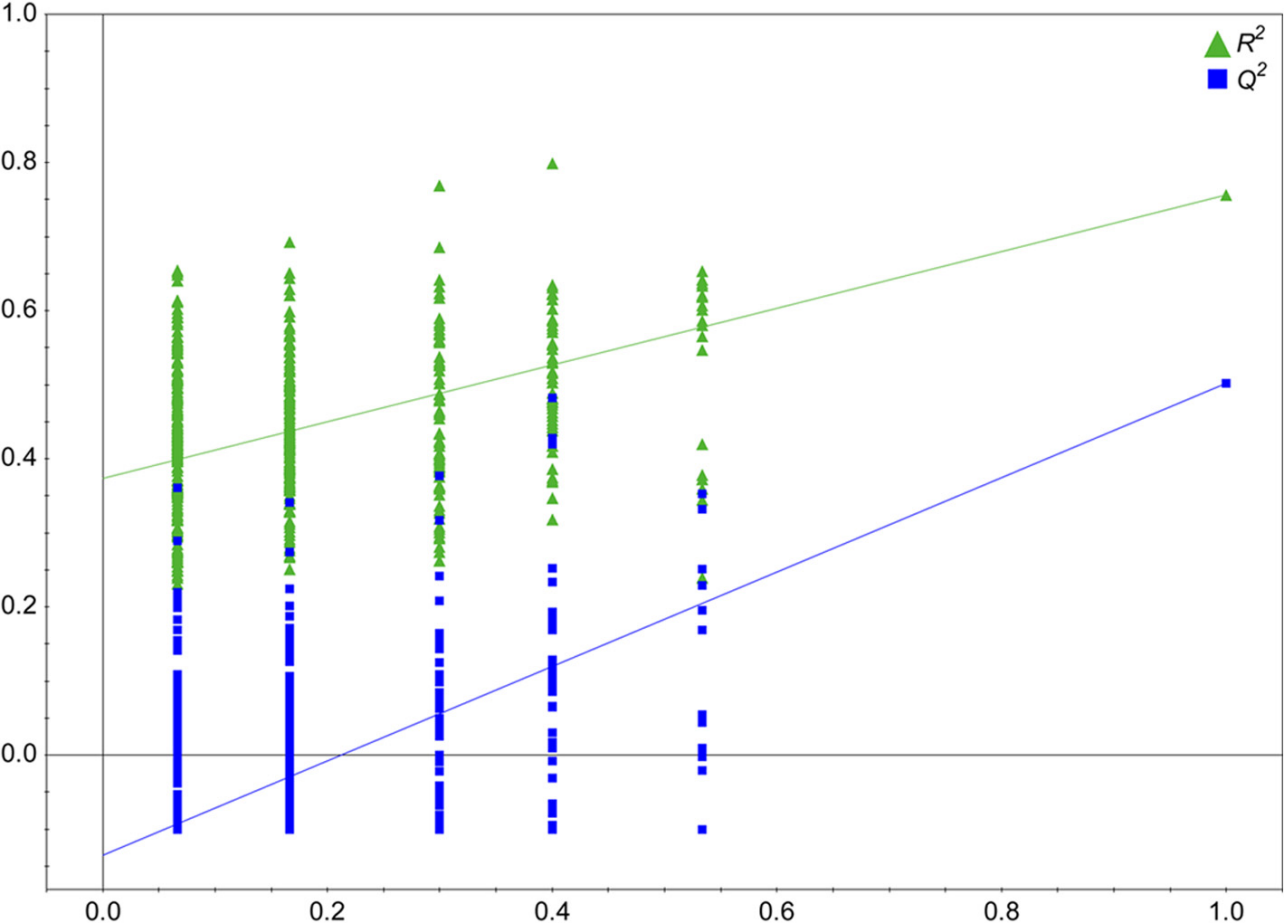
Validation plot the OPLS-DA obtained from 500 permutation tests. Y-axis intercepts of *R* ^*2*^ and *Q* ^*2*^ were 0.375 and −0.147, respectively, indicating that the original model was valid since the intercept of *R* ^*2*^ was lower than 0.4 and intercept of *Q* ^*2*^ was lower than 0.05.

The OPLS-DA model using 500 different model permutations was statistically validated. In permutation analysis, the intercept values of *R* ^*2*^ and *Q* ^*2*^ were 0.375 and −0.147, respectively. The results indicated that the OPLS-DA model of this study was strongly validated without over-fitting of the original model. It is because all permutations of the *R* ^*2*^ and *Q* ^*2*^ values to the left were lower than the original points to the right and the regression lines of the *R* ^*2*^ and *Q* ^*2*^-points intersected the vertical axis below 0.4 and 0.05, respectively (Fig 2) [20]. After employing multiple statistical analyses as described earlier, glutamate, citramalate, and valine were selected as putative biomarkers of BD with arthritis.

**Fig 3:**
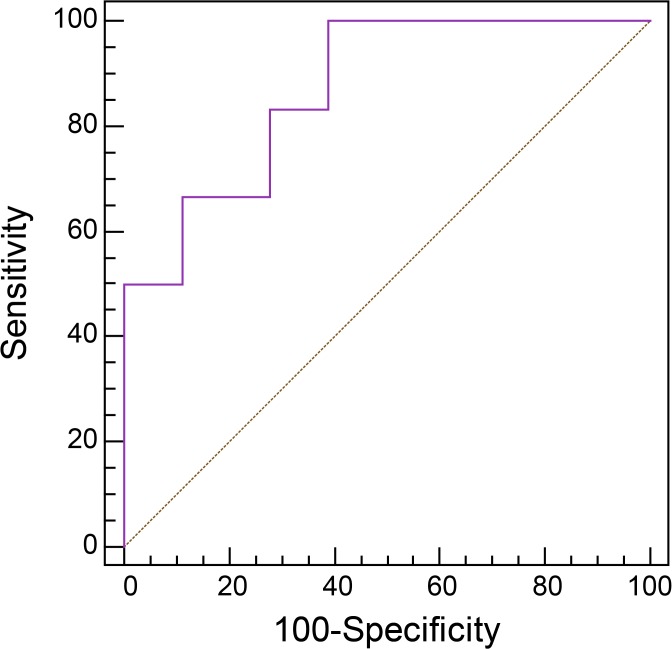
Receiver operating characteristic (ROC) curve of 3 combined biomarkers for distinguishing Behcet’s disease (BD) with arthritis from seronegative arthritis (SNA) groups. Glutamate, citramalate, and valine were selected and validated as putative biomarkers for BD with arthritis for distinguishing BD with arthritis from SNA groups by ROC curve analysis. The sensitivity and specificity were 100% and 61.1%, respectively, and the value of the area under curve (AUC) was 0.870.

Prior to evaluating the clinical utility of the 3 putative biomarkers, validation of the biomarkers was performed. ROC curves with AUC analyses, a graphical plot of sensitivity against 1-specificity, provide a numerical value for the disease diagnosis. An AUC value close to 1 indicates higher accuracy for the diagnostic test. Sensitivity of 100% and specificity of 61.1% were obtained from the ROC curve of the 3 combined biomarkers from the BD with arthritis group for discriminating BD with arthritis from SNA. The value of the AUC was 0.870, in which the 95% confidence interval was 0.671–0.971 (Fig 3). Since the 3 combined putative biomarkers showed an AUC value greater than 0.8, they were selected as biomarkers of BD with arthritis able to discriminate BD with arthritis from SNA.

## Discussion

In this study, from a total of 123 metabolites identified from SF of patients with BD with arthritis and SNA, 11 metabolites were selected as potential biomarkers through extensive statistical analyses. The level of these metabolites was significantly increased in patients with BD with arthritis compared to the SNA group (Table 2). Of those, the three most important metabolites responsible for discriminating BD with arthritis from SNRA and SNSpA were selected and validated: glutamate, citramalate, and valine. Since these three metabolites showed great discriminatory potential to distinguish BD with arthritis from SNA in this study, these metabolites are suggested to be potential biomarkers of BD with arthritis against SNA.

SF metabolomic study may contribute to a better understanding of BD with arthritis. Determining the exact roles of each metabolite was not feasible based on this data. However, we can give an explanation as to why certain metabolites are expressed differentially through literature review. Our data showed different expression of glutamate, branched chain amino acids (BCAA: valine, leucine, and isoleucine), citramalate, and methionine sulfoxide in SF of BD patients with arthritis compared to those with SNA. Clinical studies have demonstrated very high concentrations of glutamate in SF of patients with active arthritis [21]. It was reported that the astrocytes in the adult mammalian brain induce glutamate release mediated by HMGB1 [22], whose expression was significantly increased in BD [23]. These findings may explain the reason for an elevation of glutamate in BD with arthritis. Previous studies have identified changes in BCAA levels with inflammatory states such as sepsis and RA [24,25]. The level of valine was also up-regulated in adjuvant-induced arthritis rats [26]. An increased level of BCAA leads to increased production of IL-1 and/or TNF-α, which are usually elevated in RA and SNSpA [27]. From our data, it is difficult to determine the exact reason why there was an increased abundance of glutamate and BCAA in BD with arthritis compared to in SNA. Elevated expression of citramalate was found in the cerebrospinal fluid of patients with bacterial meningitis [28], indicating perturbed metabolism of glutamate in the setting of active inflammation. It is assumed that these changes are reflective of severe inflammatory changes in BD patients with arthritis compared to those with SNA, although the biological and physiological effects of glutamate, BCAA, and citramalate on inflammation and immune function are not fully understood.

Of note, elevated expression of citrulline and methionine sulfoxide was found in the SF of BD patients with arthritis in this study. Citrulline is produced from arginine as a by-product of the reaction catalyzed by the nitric oxide synthase (NOS) family. NOS activities in BD patients were significantly higher than those of the control group [29]. Oxidation of sulfur of methionine results in methionine sulfoxide or methionine sulfone by several types of reactive oxygen species (ROS) and reactive nitrogen species [30]. Neutrophils secrete large quantities of powerful oxidants at sites of inflammation and ROS produced by neutrophils may be related to the pathogenesis of BD [31]. Increased oxidative stress in BD has been well documented in previous reports [29,31]. Activated human neutrophils oxidized methionine to methionine sulfoxide [32]. Increased methionine sulfoxide in BD with arthritis is likely to be seen during times of increased oxidative stress due to the enhanced activity of neutrophils. Collectively, high expression of methionine sulfoxide and citrulline levels in BD may reflect neutrophil hyperactivity documented in BD and also suggests that SF metabolomic profiling may be useful for further understanding of the pathogenesis of BD with arthritis.

This study has some limitations mostly stemming from its small sample size and retrospective design. The most reliable method of testing the relevance of metabolomics findings is to collect new data, repeat the whole analysis and check whether the results are the same in an entirely different patient cohort. Our study had too few samples to follow this strategy. In demographic data, there was no significant difference between BD group with arthritis (34.8 ± 16.4) and SNA group including SNSpA and SNRA (40.6 ± 19.2) with respect to the patients' age. However, there was an issue in the difference of gender ratio between two groups. Differences in age and gender ratios represented in comparative groups are potentially confounders of metabolism. It has been reported that there are age- and gender-dependent alternations in human plasma [33]. On the other hand, the metabolic profiles were not significantly affected by gender in the plasma of patients with RA [34]. Although the OPLS-DA model was well validated by the results obtain from permutation tests at the present stage, validation of confounding factors still remains a significant challenge in most metabolomics studies.

In summary, we have found distinct metabolic differences and potential biomarkers for discriminating BD with arthritis from SNA. This is the first report seeking to identify potential SF biomarkers for differentiating between BD with arthritis and SNA and to understand the mechanism of BD with arthritis using an analysis of metabolic perturbations. Further studies including larger patients and control cohorts are warranted to validate metabolite profiles for clinical use and to ascertain the feasibility of metabolomic analysis to provide new perspectives for the characterization of different clinical phenotypes of BD.

## Supporting Information

S1 Fig PCA score plot of the metabolite profiles of Behcet’s disease with arthritis (BD) and seronegative arthritis (SNA) groups. The metabolite profiles showed slight discrimination between BD and SNA groups. The generated explained variation values, 0.40 of *R* ^*2*^ *X* and the predictive capability, 0.41 of *Q* ^*2*^.
(TIF)

S2 Fig Fold changes of absolute concentrations of 3 metabolites in synovial fluid selected as potential biomarkers for Behcet’s disease with arthritis. These metabolites increased in Behcet’s disease with arthritis group compared to seronegative arthritis group.
(TIF)

S1 Table The list of the selected metabolites with VIP values more than 1. (DOCX)

## References

[1] Sakane T, Takeno M, Suzuki N, Inaba G. Behcet's disease. N Engl J Med. 1999;341:1284–1291. 10528040 10.1056/NEJM199910213411707

[2] Bicer A. Musculoskeletal Findings in Behcet's Disease. Patholog Res Int. 2012;2012:653806 10.1155/2012/653806 21961082 PMC3180072

[3] Nicholson JK, Holmes E, Kinross JM, Darzi AW, Takats Z, Lindon JC. Metabolic phenotyping in clinical and surgical environments. Nature. 2012;491:384–392. 10.1038/nature11708 23151581

[4] Yurdakul S, Yazici H, Tuzun Y, Pazarli H, Yalcin B, Altac M, et al The arthritis of Beh?et's disease: a prospective study. Ann Rheum Dis. 1983;42:505–515. 6625699 10.1136/ard.42.5.505 PMC1001284

[5] Hatemi G, Fresko I, Tascilar K, Yazici H. Increased enthesopathy among Behcet's syndrome patients with acne and arthritis: an ultrasonography study. Arthritis Rheum. 2008;58:1539–1545. 10.1002/art.23450 18438817

[6] Frikha F, Marzouk S, Kaddour N, Frigui M, Bahloul Z. Destructive arthritis in Behcet's disease: a report of eight cases and literature review. Int J Rheum Dis. 2009;12:250–255. 10.1111/j.1756-185X.2009.01419.x 20374355

[7] Gibson T, Laurent R, Highton J, Wilton M, Dyson M, Millis R. Synovial histopathology of behcet's syndrome. Ann Rheum Dis. 1981;40:376–381. 7020613 10.1136/ard.40.4.376 PMC1000732

[8] Vernon-Roberts B, Barnes CG, Revell PA. Synovial pathology in Behcet's syndrome. Ann Rheum Dis. 1978;37:139–145. 646465 10.1136/ard.37.2.139 PMC1001179

[9] Kaddurah-Daouk R, Kristal BS, Weinshilboum RM. Metabolomics: a global biochemical approach to drug response and disease. Annu Rev Pharmacol Toxicol. 2008;48:653–683. 10.1146/annurev.pharmtox.48.113006.094715 18184107

[10] Fitzpatrick M, Young SP. Metabolomics-a novel window into inflammatory disease. Swiss Med Wkly. 2013;143:w13743 10.4414/smw.2013.13743 23348753 PMC4337982

[11] Priori R, Scrivo R, Brandt J, Valerio M, Casadei L, Valesini G, et al Metabolomics in rheumatic diseases: the potential of an emerging methodology for improved patient diagnosis, prognosis, and treatment efficacy. Autoimmun Rev. 2013;12:1022–1030. 10.1016/j.autrev.2013.04.002 23688955

[12] Scrivo R, Casadei L, Valerio M, Priori R, Valesini G, Manetti C. Metabolomics approach in allergic and rheumatic diseases. Curr Allergy Asthma Rep. 2014;14:445 10.1007/s11882-014-0445-5 24744271

[13] International Study Group for Behcet's Disease. Criteria for diagnosis of Behcet's disease. International Study Group for Behcet's Disease. Lancet. 1990;335:1078–1080. 1970380

[14] Rudwaleit M, van der Heijde D, Landewe R, Listing J, Akkoc N, Brandt J, et al The development of Assessment of SpondyloArthritis international Society classification criteria for axial spondyloarthritis (part II): validation and final selection. Ann Rheum Dis. 2009;68:777–783. 10.1136/ard.2009.108233 19297344

[15] Arnett FC, Edworthy SM, Bloch DA, McShane DJ, Fries JF, Cooper NS, et al The American Rheumatism Association 1987 revised criteria for the classification of rheumatoid arthritis. Arthritis Rheum. 1988;31:315–324. 3358796 10.1002/art.1780310302

[16] Kim S, Hwang J, Xuan J, Jung YH, Cha HS, Kim KH. Global metabolite profiling of synovial fluid for the specific diagnosis of rheumatoid arthritis from other inflammatory arthritis. PLOS ONE. 2014;9:e97501 10.1371/journal.pone.0097501 24887281 PMC4041724

[17] Kim S, Lee DY, Wohlgemuth G, Park HS, Fiehn O, Kim KH. Evaluation and optimization of metabolome sample preparation methods for *Saccharomyces cerevisiae* . Anal Chem. 2013;85:2169–2176. 10.1021/ac302881e 23289506

[18] Lee DY, Fiehn O. High quality metabolomic data for *Chlamydomonas reinhardtii* . Plant Methods. 2008;4:7 10.1186/1746-4811-4-7 18442406 PMC2377246

[19] Fiehn O, Wohlgemuth G, Scholz M, Kind T, Lee DY, Lu Y, et al Quality control for plant metabolomics: reporting MSI-compliant studies. Plant J. 2008;53:691–704. 10.1111/j.1365-313X.2007.03387.x 18269577

[20] Umetrics AB. User's Guide to SIMCA-P, SIMCA-P+ version 11.0. Sweden: Umetrics AB: Umea° 2005.

[21] McNearney T, Baethge BA, Cao S, Alam R, Lisse JR, Westlund KN. Excitatory amino acids, TNF-alpha, and chemokine levels in synovial fluids of patients with active arthropathies. Clin Exp Immunol. 2004;137:621–627. 15320917 10.1111/j.1365-2249.2004.02563.x PMC1809131

[22] Bonanno G, Raiteri L, Milanese M, Zappettini S, Melloni E, Pedrazzi M, et al The high-mobility group box 1 cytokine induces transporter-mediated release of glutamate from glial subcellular particles (gliosomes) prepared from in situ-matured astrocytes. Int Rev Neurobiol. 2007;82:73–93. 17678956 10.1016/S0074-7742(07)82004-6

[23] Ahn JK, Cha HS, Bae EK, Lee J, Koh EM. Extracellular high-mobility group box 1 is increased in patients with Behcet's disease with intestinal involvement. J Korean Med Sci. 2011;26:697–700. 10.3346/jkms.2011.26.5.697 21532866 PMC3082127

[24] Freund HR, Ryan JA Jr., Fischer JE. Amino acid derangements in patients with sepsis: treatment with branched chain amino acid rich infusions. Ann Surg. 1978;188:423–430. 99098 10.1097/00000658-197809000-00017 PMC1396972

[25] Trang LE, Furst P, Odeback AC, Lovgren O. Plasma amino acids in rheumatoid arthritis. Scand J Rheumatol. 1985;14:393–402. 4081662 10.3109/03009748509102044

[26] Qi Y, Li S, Pi Z, Song F, Lin N, Liu S, et al Metabonomic study of Wu-tou decoction in adjuvant-induced arthritis rat using ultra-performance liquid chromatography coupled with quadrupole time-of-flight mass spectrometry. J Chromatogr B Analyt Technol Biomed Life Sci. 2014;953–954:11–19. 10.1016/j.jchromb.2014.01.044 24561351

[27] Bassit RA, Sawada LA, Bacurau RF, Navarro F, Martins E Jr., Santos RV, et al Branched-chain amino acid supplementation and the immune response of long-distance athletes. Nutrition. 2002;18:376–379. 11985939 10.1016/s0899-9007(02)00753-0

[28] Perlman S, Carr SA. Citramalic acid in cerebrospinal fluid of patients with bacterial meningitis. Clin Chem. 1984;30:1209–1212. 6145530

[29] Taysi S, Sari RA, Dursun H, Yilmaz A, Keles M, Cayir K, et al Evaluation of nitric oxide synthase activity, nitric oxide, and homocysteine levels in patients with active Behcet's disease. Clin Rheumatol. 2008;27:1529–1534. 10.1007/s10067-008-0963-4 18636307

[30] Levine RL, Moskovitz J, Stadtman ER. Oxidation of methionine in proteins: roles in antioxidant defense and cellular regulation. IUBMB Life. 2000;50:301–307. 11327324 10.1080/713803735

[31] Pineton de Chambrun M, Wechsler B, Geri G, Cacoub P, Saadoun D. New insights into the pathogenesis of Behcet's disease. Autoimmun Rev. 2012;11:687–698. 10.1016/j.autrev.2011.11.026 22197900

[32] Tsan MF, Chen JW. Oxidation of methionine by human polymorphonuclear leukocytes. J Clin Invest. 1980;65:1041–1050. 6245104 10.1172/JCI109756 PMC371434

[33] Lawton KA, Berger A, Mitchell M, Milgram KE, Evans AM, Guo L, et al Analysis of the adult human plasma metabolome. Pharmacogenomics. 2008;9:383–397. 10.2217/14622416.9.4.383 18384253

[34] Lauridsen MB, Bliddal H, Christensen R, Danneskiold-Samsoe B, Bennett R, Keun H, et al 1H NMR spectroscopy-based interventional metabolic phenotyping: a cohort study of rheumatoid arthritis patients. J Proteome Res. 2010;9:4545–4553. 10.1021/pr1002774 20701312

