# Supplementary figures and images for: A Comparative Metabolomic Evaluation of Behcet’s Disease with Arthritis and Seronegative Arthritis Using Synovial Fluid
Source: PLoS One. 2015 Aug 13;10(8):e0135856. doi: 10.1371/journal.pone.0135856 (PMC4536180; doi:10.1371/journal.pone.0135856)

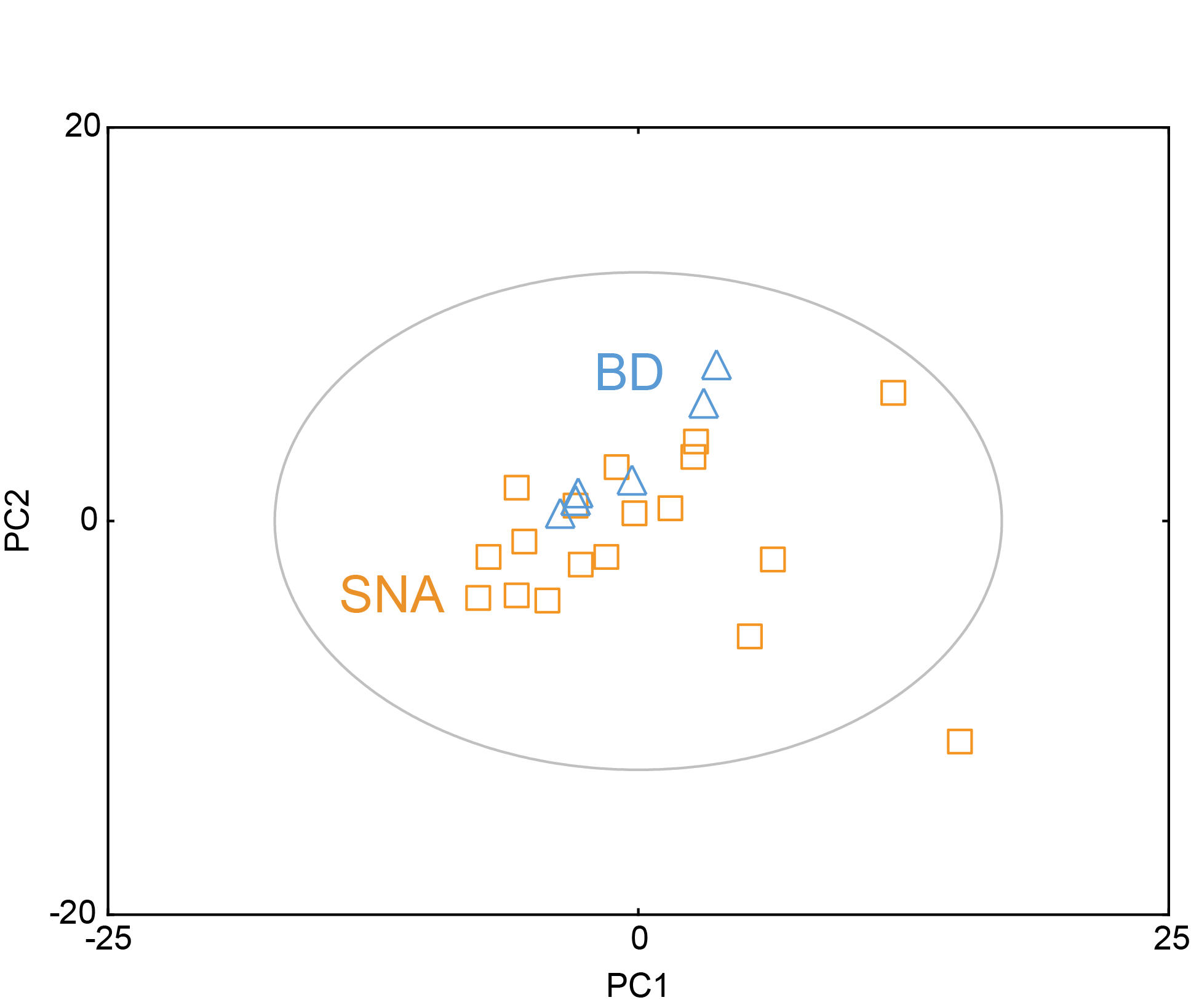

Supplement: S1 Fig — The metabolite profiles showed slight discrimination between BD and SNA groups. The generated explained variation values, 0.40 of R 2 X and the predictive capability, 0.41 of Q 2. (TIF) [file pone.0135856.s001.tif]

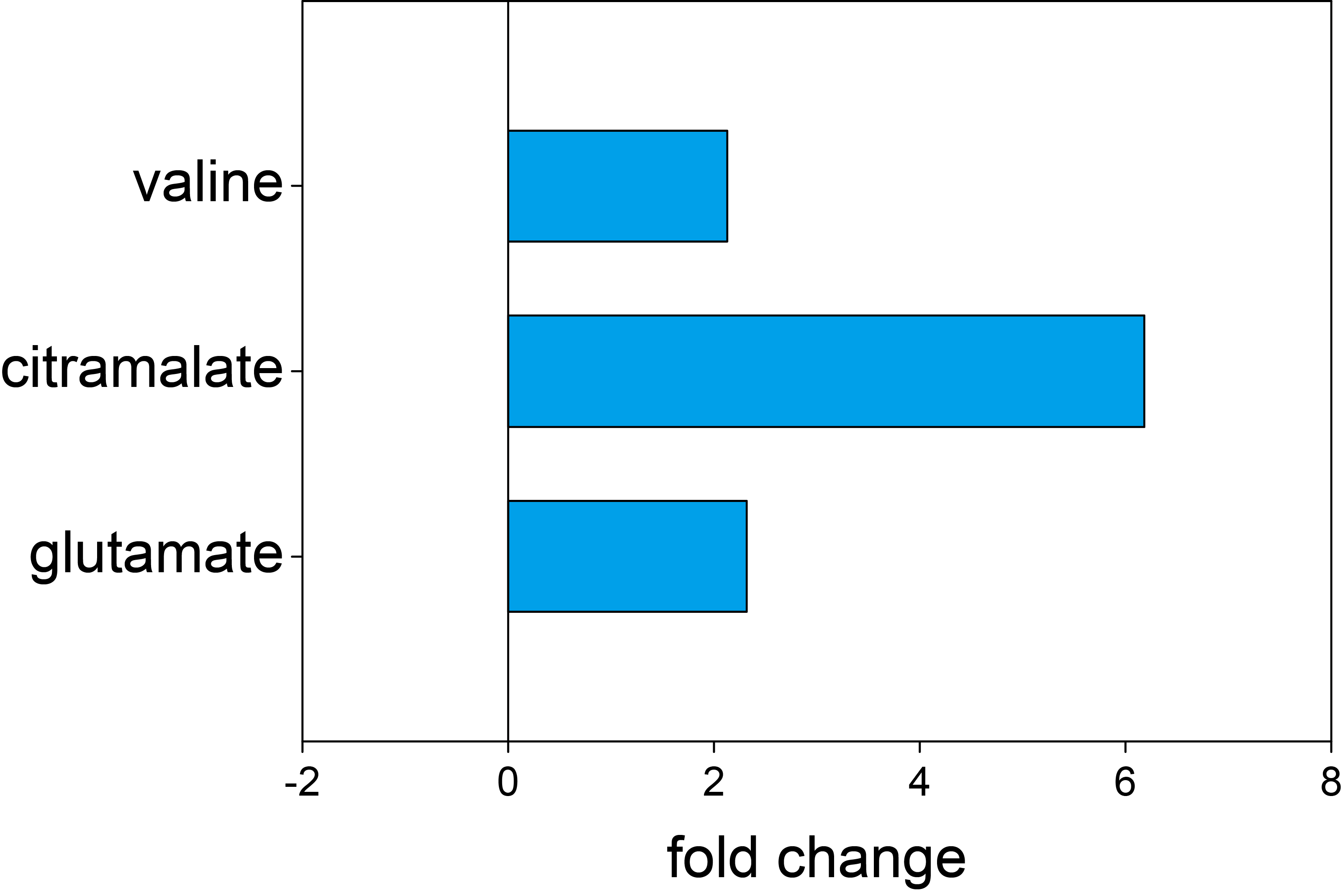

Supplement: S2 Fig — These metabolites increased in Behcet’s disease with arthritis group compared to seronegative arthritis group. (TIF) [file pone.0135856.s002.tif]
