# Supplementary material for: A Comparative Metabolomic Evaluation of Behcet’s Disease with Arthritis and Seronegative Arthritis Using Synovial Fluid
Source: PLoS One. 2015 Aug 13;10(8):e0135856. doi: 10.1371/journal.pone.0135856 (PMC4536180; doi:10.1371/journal.pone.0135856)
Supplement: S1 Table — (DOCX) [file pone.0135856.s003.docx]

| **Metabolite** | **VIP** | **Metabolite** | **VIP** |
| --- | --- | --- | --- |
| glutamate | 2.68 | pyruvate | 1.13 |
| citramalate | 2.55 | phosphogluconate | 1.08 |
| valine | 2.34 | citrate | 1.04 |
| leucine | 2.18 | fructose | 1.03 |
| methionine sulfoxide | 2.17 | serine | 1.55 |
| glycerate | 2.16 | palmitic acid | 1.53 |
| phosphate | 2.08 | glycerol-1-phosphate | 1.27 |
| lysine | 2.01 | 3,6-anhydro-D-galactose | 1.04 |
| isoleucine | 1.93 | octadecanol | 1.31 |
| urea | 1.85 | palmitoleic acid | 1.07 |
| citrulline | 1.77 | ornithine | 1.21 |
| methionine | 1.72 | aspartate | 1.17 |
| glutamine | 1.70 | arachidic acid | 1.10 |
| α-tocopherol | 1.64 | trehalose | 1.42 |
| stearic acid | 1.62 | glycerol | 1.22 |
| oxoproline | 1.58 | putrescine | 1.33 |
| adenosine | 1.30 | cellobiose | 1.32 |
| histidine | 1.17 | arabitol | 1.01 |
| tagatose | 1.15 |  |  |
